# Conserved behavioral and genetic mechanisms in the pre-hatching molt of the nematode *Pristionchus pacificus*

**DOI:** 10.1186/2041-9139-5-31

**Published:** 2014-09-19

**Authors:** Victor M Lewis, Ray L Hong

**Affiliations:** 1 Department of Biology, California State University, Northridge, 18111 Nordhoff Street, Northridge, CA 91330-8303, USA

**Keywords:** Molting, Ecdysis, Developmental timing, Nematode, Heterochrony

## Abstract

**Background:**

During development, juvenile nematodes undergo four molts. Although the number of molts appears to be constant within the Nematoda, the timing of the first molt can occur either before or after hatching. A previous study indicates that, as in some parasitic nematode lineages, a pre-hatching juvenile stage also exists in Diplogastrid nematodes. A detailed description of these sequence of events has yet to be shown for any single species.

**Findings:**

To delineate the timing of the pre-hatching molt in the beetle-associated *Pristionchus pacificus*, we tracked individual mid-J1 stage worms inside the eggshell through the J1-J2 transition and hatching. We found that active movement ended 21 hours after egg-laying, followed by lethargus and hatching. We inferred that lethargus behavior represents the onset of the first molt, which precedes each post-hatching molt in *C. elegans* and *P. pacificus*. The onset of the J1-J2 molt was also marked by the upregulation of the *P. pacificus* molting marker *Ppa-pnhr-1*. We further corroborated the pre-hatching molt with the isolation of two genetic mutants that exhibited aberrant molting both inside the egg and after hatching, as characterized by protracted and often-aborted shedding of the old cuticle.

**Conclusion:**

Our results describe in detail the pre-hatching juvenile molt in *P. pacificus*, provide strong visual evidence of a pre-hatching molt, and show support for common genetic mechanisms regulating molting in the pre-hatching and post-hatching developmental stages. Our findings support the hypothesis that the evolution of pre-hatching development in Diplogastrid nematodes is likely due to a heterochronic shift between the timing of the first molt and hatching.

## Findings

### Introduction

The Ecdysozoa is a clade of invertebrates, including nematodes and arthropods, which share the common process of shedding the exoskeleton or cuticle. The nematode cuticle is a multi-layered extracellular matrix that protects the entire body of the growing organism [1]. During transitions from one juvenile stage to another, the new cuticle emerges underneath and eventually replaces the old cuticle to accommodate the larger body of the next developmental stage. Molting starts with apolysis, or shedding of the old cuticle, followed by synthesis of the new cuticle, and culminates with ecdysis, or escape from the old cuticle. Nematodes undergo molting transition from active foraging behavior to lethargus, a quiescent period marked by a lack of movement during which the new cuticle is synthesized. The end of lethargus and the resumption of active behavior occur just prior to ecdysis, when the worm breaches the old cuticle [2].

Unlike arthropods, which can vary in both the timing and number of molts, all nematodes during larval development undergo four molts before reaching reproductive maturity [3]. This phylum-wide developmental constraint is likely not limited by genetic restrictions as single gene mutations in the model nematode *Caenorhabditis elegans* can cause changes to the molting cycle, such as supernumerary molts in *lin-4*, *lin-14*, and *lin-29* mutants [4,5]. Although the four molts after hatching in *C. elegans* appear to be the ancestral phenotype, there are several examples that deviate from this pattern in plant and animal parasites [6]. Nematodes that molt prior to hatching include the parasitic pinewood nematode *Bursaphelenchus xylophilus* and the potato cyst nematode *Globodera pallida*[3,7,8], as well as the vertebrate parasites *Ascaris lumbricoides* and *Toxocara cati*[3,9]*.* Among non-parasitic nematodes, larvae that undergo the first molt within the eggshell have been described in the *Pristionchus* and *Oigolaimella* species in the family Diplogastridae [10]. Because the evolution of parasitism in nematodes may involve stepwise changes between free-living, phoretic, necromenic, and parasitic lifestyles [11,12], the *P. pacificus* necromenic lifestyle in close association with beetles invites studies on developmental plasticity, timing, and novelty. In particular, the presence of the pre-hatching molt within these diverse lineages hints at the role a pre-hatching larval stage may play in driving the evolution of parasitism.

*P. pacificus* was once thought to represent a unique group that possess only three juvenile stages prior to adulthood [6]. Subsequent investigation has since revealed that the first molt actually occurs prior to hatching in Diplogastrid nematodes, including *P. pacificus*[10]. Behavioral events that characterize this as a complete developmental stage have not been observed, but this observation is crucial for the understanding of developmental timing. In order to establish behavioral and genetic similarities between the pre-hatching and post-hatching molts in *P. pacificus*, we tracked development from late-stage embryos through the stage 1 juvenile (J1) to J2 transition and hatching. We further corroborated our observations by monitoring expression changes of a *P. pacificus* molting marker as well as obtaining general molting defective mutants that show both pre-hatching and post-hatching molting-defects. Our findings further support the hypothesis that the pre-hatching J1 stage in *P. pacificus* is homologous to the first post-hatching L1 stage in *C. elegans*.

### Results

**Figure 1:**
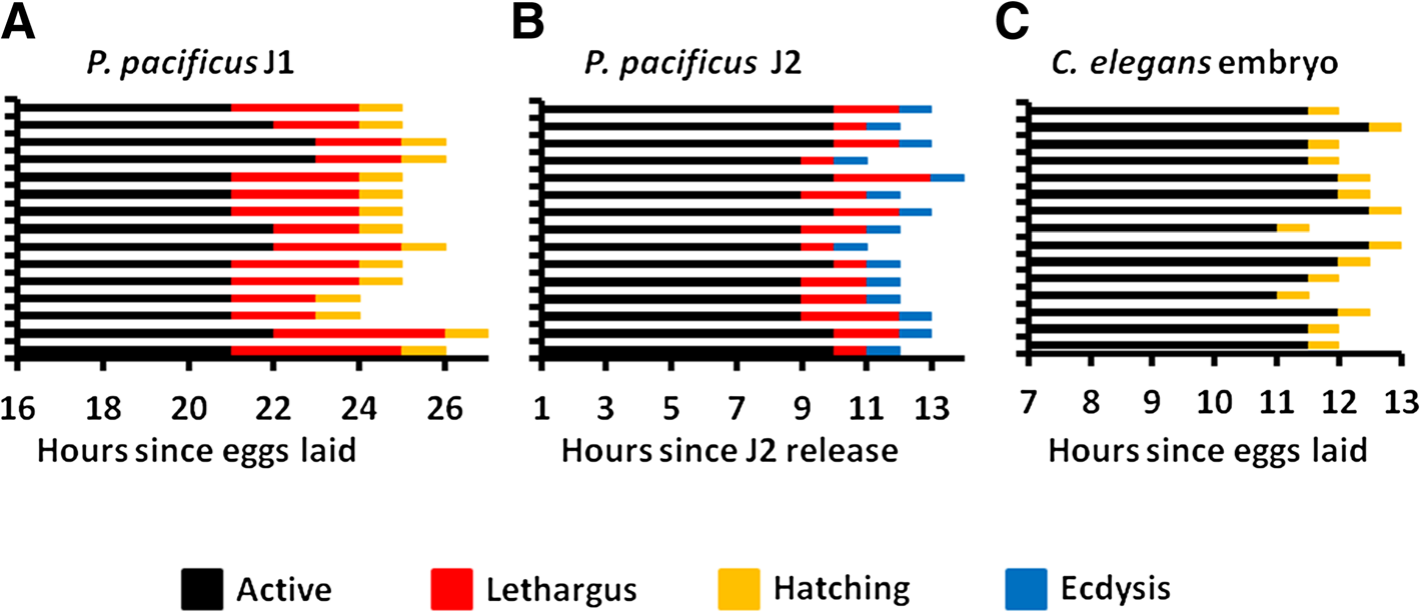
**Pre-hatching juvenile (J1) development in *P. pacificus*. (A)** Pre-hatching development in wild-type *P. pacificus* PS312. **(B)** Post-hatching development in J2 stage wild-type *P. pacificus*. The sequence of behaviors observed in **(A)** is almost identical to those in **(B)**. **(C)** Late-embryonic development in wild-type *C. elegans* N2 lacks a lethargus period.

To delineate the sequence of events leading to the first molt in *P. pacificus* eggs*,* we tracked development from the middle of the first J1 juvenile stage until hatching in individual worms (n = 15). For convenience, we presumed the end of embryogenesis begins around 13 hours when the worm ceases to elongate and J1 cuticle synthesis is superficially complete [10,13]. Because molting is a dynamic process that is difficult to pinpoint directly using a single morphological feature, especially inside the confined space within the eggshell, we specifically looked for developmental lethargus that typically indicates the onset of molt in *C. elegans.* From 16 hours on, worms were observed for 10 minutes once every hour using Differential Interference Contrast (DIC) microscopy. Worms were considered active when movement occurred consistently throughout the 10-minute window, and in lethargus when there was minimal movement during the same time frame. Hatching was confirmed visually when the worm leaves its eggshell. We inferred that ecdysis occurred between the termination of lethargus and the onset of hatching, in rapid succession. Active behavior during the J1 stage of development ended 21.5 ± 0.74 hours post egg-laying, followed by lethargus 24.1 ± 0.64 hours post egg-laying, and hatching 25.1 ± 0.64 post egg-laying (Figure 1A; Additional file 1). Active behavior consisted of rapid movements, including full-body turns, interspaced by short periods of no movement. In contrast, worms in lethargus remained relatively motionless, with only slight repositioning movements during the approximately 2.5-hour lethargus period. Hatching was characterized by rapid turning movements that culminated with ramming of the eggshell within one hour of the end of lethargus. Therefore, lethargus coincides with the timing of the pre-hatching molt similar to the molting behavior found in *C. elegans.*

To determine if the observed pre-hatching behaviors were similar to the behaviors associated with molting in post-hatching *P. pacificus*, we tracked an additional population of worms from the post-hatching J2 arrest through the J2-J3 molt. Active behavior ended 9.5 ± 0.51 hours after J2 release on food, followed by lethargus after 11.4 ± 0.81 hours, and ecdysis after 12.4 ± 0.81 hours (Figure 1B). Active behavior consisted of typical feeding activity interspaced by short rests. Lethargus behavior was similarly characterized by a general lack of movement, although short movements, totaling no more than one body length, were occasionally observed (Additional file 2). Worms undergoing ecdysis were observed to resume activity with quick longitudinal movement coupled with whole-body turns, presumably to dislodge the previous cuticle. Thus, the post-hatching J2-J3 molt also exhibits the characteristic lethargus state found in the earlier J1-J2 molt inside the egg. To confirm that our observation is indeed restricted to Diplogastrid nematodes, we also tracked a population of *C. elegans* embryos from post-elongation embryogenesis through hatching. Active behavior during late-embryogenesis ended 11.8 ± 0.50 hours after egg laying and hatching occurred 12.3 ± 0.52 hours after egg laying (Figure 1C; Additional file 3). No lethargus period was observed. We also did not observe a possible molt inside the *C. elegans* eggshell that was reported to be limited to the pharyngeal cuticle [2]. In conclusion, we observed the same stereotyped behavioral events leading to a J1-J2 molt in the late-stage pre-hatching *P. pacificus* juveniles as those observed in newly hatched *P. pacificus* J2 juveniles and the molts in *C. elegans*.

**Figure 2:**
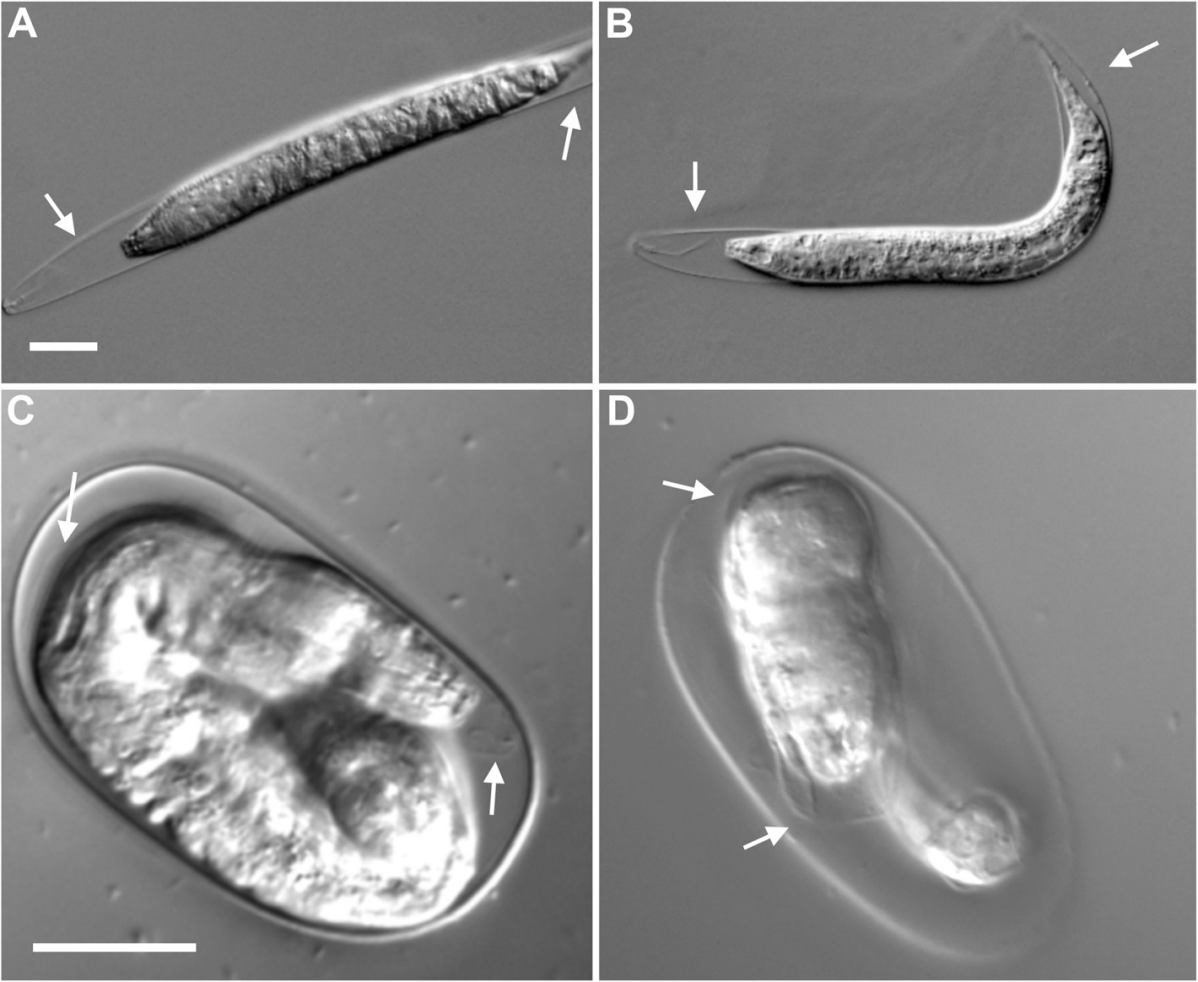
**Molting-deficient phenotype of *Ppa-mlt *mutants.** Post-hatching stage 2 juvenile (J2) to J3 molt **(A)***Ppa-mlt(csu26)*, and **(B)***Ppa-mlt(csu28)*. Pre-hatching molt **(C)***Ppa-mlt(csu26),* and **(D)***Ppa-mlt(csu28)*. White arrows indicate unshed cuticle from previous stage. The unshed cuticle in the mutants can be observed in the posterior **(A, B)** and mid-body **(C, D)** regions *-* a phenotype not observed in wild-type. Scale bars = 20 μm.

To provide additional evidence that molting occurs inside the eggshell, we performed a genetic screen for general molting-defective mutants. We surmised that post-hatching molting-deficient mutants would also include eggs with deficiencies in the pre-hatching molt, given that many genetic regulators affect all molts [14]. We performed a non-saturating genetic screen for *P. pacificus* F_2_ juveniles with defective molting and isolated two complementing mutant strains, *Ppa-mlt(csu26)* and *Ppa-mlt(csu28)*. The molting deficiency is easily identified by the intact shed cuticle surrounding both the anterior and posterior parts of the worm, whereas the cuticle of wild-type worms protrude only from the anterior end prior to ecdysis (Figure 2; Additional file 4). To assess the possibility of heterochronic changes in our mutant lines, we tracked *Ppa-mlt(csu26)* and *Ppa-mlt(csu28)* from a synchronized J2 stage to adulthood and observed that although the time required to reach adulthood was longer in the *csu28* line, all worms progressed through every stage and no molt was repeated or skipped (Table 1). The majority of post-hatching molting defects were observed in the J2-J3 molt, which is expected of mutations in genes responsible for a general molting process (Table 2). As no newly hatched J2 worms exhibited molting deficiencies, we hypothesize that *Ppa-mlt* juveniles that displayed the molting deficiency in the eggshell were unable to properly hatch. By subtracting the number of worms that hatched from those in the J1 stage, we inferred that the frequency of the pre-hatching molting deficiency is 11.43% and 16.38% for *csu26* and *csu28,* respectively (Table 3). No wild-type worms showed pre-hatching molting defects using the same criteria.

**Table 1:** **Time to adulthood in *Ppa-mlt *** **mutants**

	**J2**	**J3**	**J4**	**Adult**		**J2**	**J3**	**J4**	**Adult**
**0 hours**					**48 hours**				
Wild-type PS312	1.00	-	-	-	Wild-type PS312	-	-	0.72	0.28
*Ppa-mlt(csu26)*	1.00	-	-	-	*Ppa-mlt(csu26)*	-	-	0.88	0.12
*Ppa-mlt(csu28)*	1.00	-	-	-	*Ppa-mlt(csu28)*	-	0.40	0.56	0.04
**12 hours**					**60 hours**				
Wild-type PS312	0.05	0.95	-	-	Wild-type PS312	-	-	-	1.00
*Ppa-mlt(csu26)*	0.13	0.87	-	-	*Ppa-mlt(csu26)*	-	-	-	1.00
*Ppa-mlt(csu28)*	0.34	0.66	-	-	*Ppa-mlt(csu28)*	-	0.25	0.64	0.11
**24 hours**					**72 hours**				
Wild-type PS312	-	0.86	0.14	-	Wild-type PS312	-	-	-	1.00
*Ppa-mlt(csu26)*	-	0.98	0.02	-	*Ppa-mlt(csu26)*	-	-	-	1.00
*Ppa-mlt(csu28)*	-	1.00	-	-	*Ppa-mlt(csu28)*	-	-	0.58	0.42
**36 hours**					**84 hours**				
Wild-type PS312	-	0.02	0.86	0.12	Wild-type PS312	-	-	-	1.00
*Ppa-mlt(csu26)*	-	0.09	0.87	0.04	*Ppa-mlt(csu26)*	-	-	-	1.00
*Ppa-mlt(csu28)*	-	0.58	0.42	-	*Ppa-mlt(csu28)*	-	-	-	1.00

Development from stage 2 juvenile (J2) arrest showing fraction per stage in PS312 (n = 55), *csu26* (n = 53), and *csu28* (n = 45) after release from J2 arrest at 12-hour intervals. Individual mutants that arrested during a molt were not included in the total count.

**Table 2:** **Frequency of molting deficient phenotype in *Ppa-mlt *** **mutants**

	**J2**	**J3**	**J4**	**Total**
Wild-type PS312	0.004	-	-	0.004
*Ppa-mlt(csu26)*	0.201	0.011	-	0.213
*Ppa-mlt(csu28)*	0.092	0.020	0.006	0.118

Stage-specific frequency of molting deficiency in PS312 (n = 1061), *csu26* (n = 800), and *csu28* (n = 500).

**Table 3:** **Pre-hatching molting-deficient phenotype in *Ppa-mlt *** **mutants**

	**Viable J1**	**Hatched**	**Inferred molt-deficient**
Wild-type PS312	0.93	0.93	0.00
*Ppa-mlt(csu26)*	0.71	0.60	0.11
*Ppa-mlt(csu28)*	0.50	0.33	0.17

The pre-hatching frequency of the molting deficient phenotype was inferred in PS312 (n = 235), *csu26* (n = 223), and *csu28* (n = 46). J1, stage 1 juvenile.

**Figure 3:**
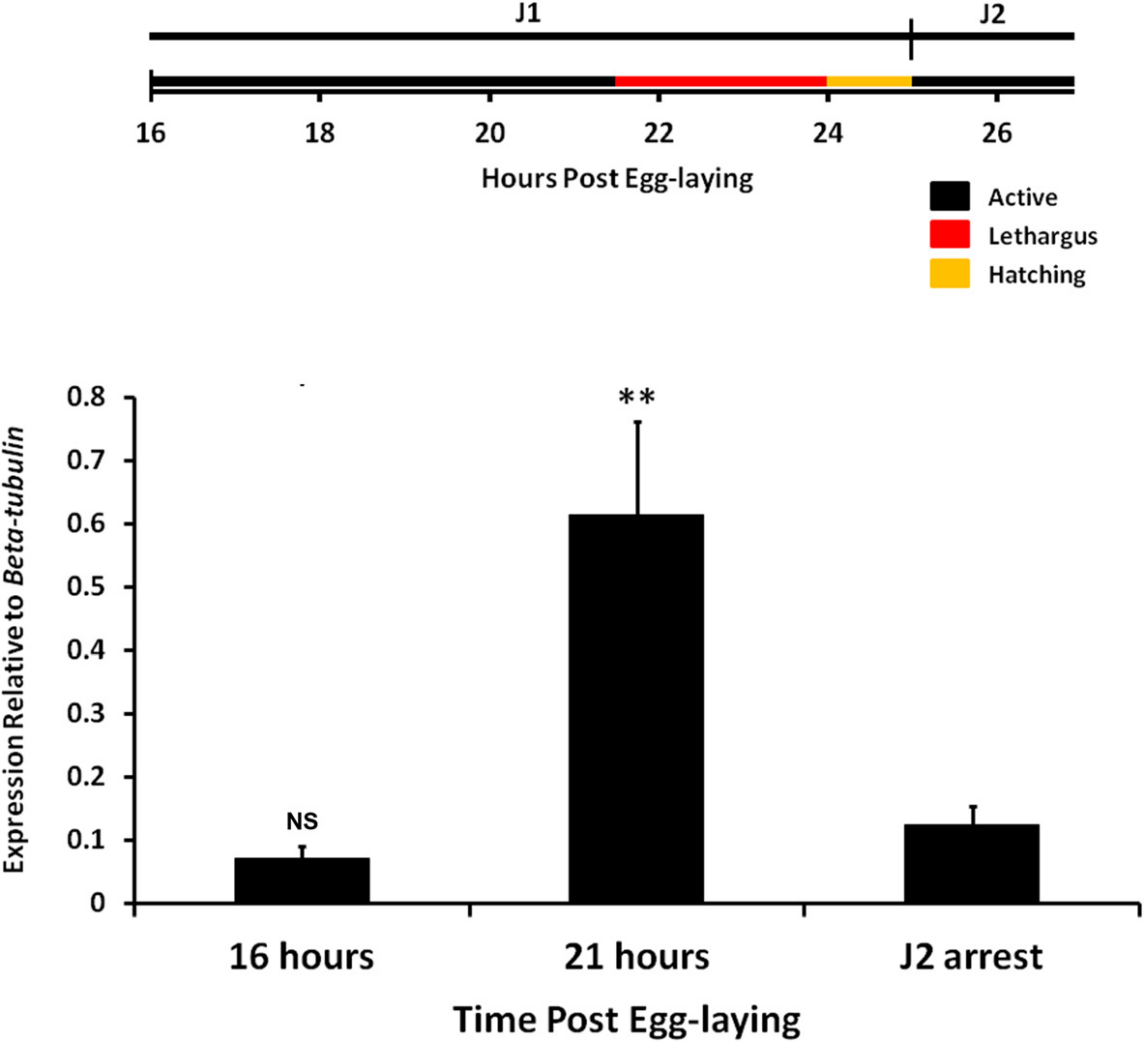
**Expression of the nuclear receptor ultraspiracle (Usp) homolog *Ppa-pnhr-1 *is expressed prior to the first molt inside the egg.** Expression is low in early stage 1 juvenile (J1) (16 hours) and high in late J1 (21 hours), indicative of a pending molt. The J2 developmental arrest serves as the negative control. Data represent eight total qPCR reactions from two independent cDNA samples at each time point. Each cDNA sample was collected from RNA from four pooled synchronized populations. The relative expression ratio of *Ppa-pnhr-1* to *Ppa-beta-tubulin* was normalized to the no-reverse transcriptase negative control. Error bars represent standard error of the mean. **P ≤0.01 (Dunn’s test to J2 arrest).

Finally, to further substantiate our observations, we looked for gene expression patterns indicative of molting. *Ppa-pnhr-*1 is a previously identified homolog of the nuclear receptor ultraspiracle (Usp), a well characterized member of the arthropod molting pathway [15-17]. *Ppa-pnhr-1* has been shown to have cyclical peaks of expression prior to all three post-hatching molts, and is the only known marker associated with the onset of molting in *P. pacificus*[18]. Using quantitative real-time PCR, we measured mRNA expression and found that *Ppa-pnhr-1* level remained low in the early J1 followed by a distinct peak prior to the J1-J2 molt (Figure 3). As a negative control, we also assayed the *Ppa-pnhr-1* expression profile at the J2 developmental arrest, a period which occurs immediately after hatching in the absence of food. During this arrested development, when we are certain that nematodes are not approaching the J2-J3 molt, the level of *Ppa-pnhr-1* expression is much lower than 21-hour embryos and not significantly different from 16-hour embryos. The timing of *Ppa-pnhr-1* peak expression coincides with our observation of lethargus shortly before the first molt inside the egg. The similarity of lethargus behavior and increased expression of the post-hatching molting marker *Ppa-pnhr-1* strongly suggests that the molt inside the egg is a bonafide molting event.

### Conclusion

**Figure 4:**
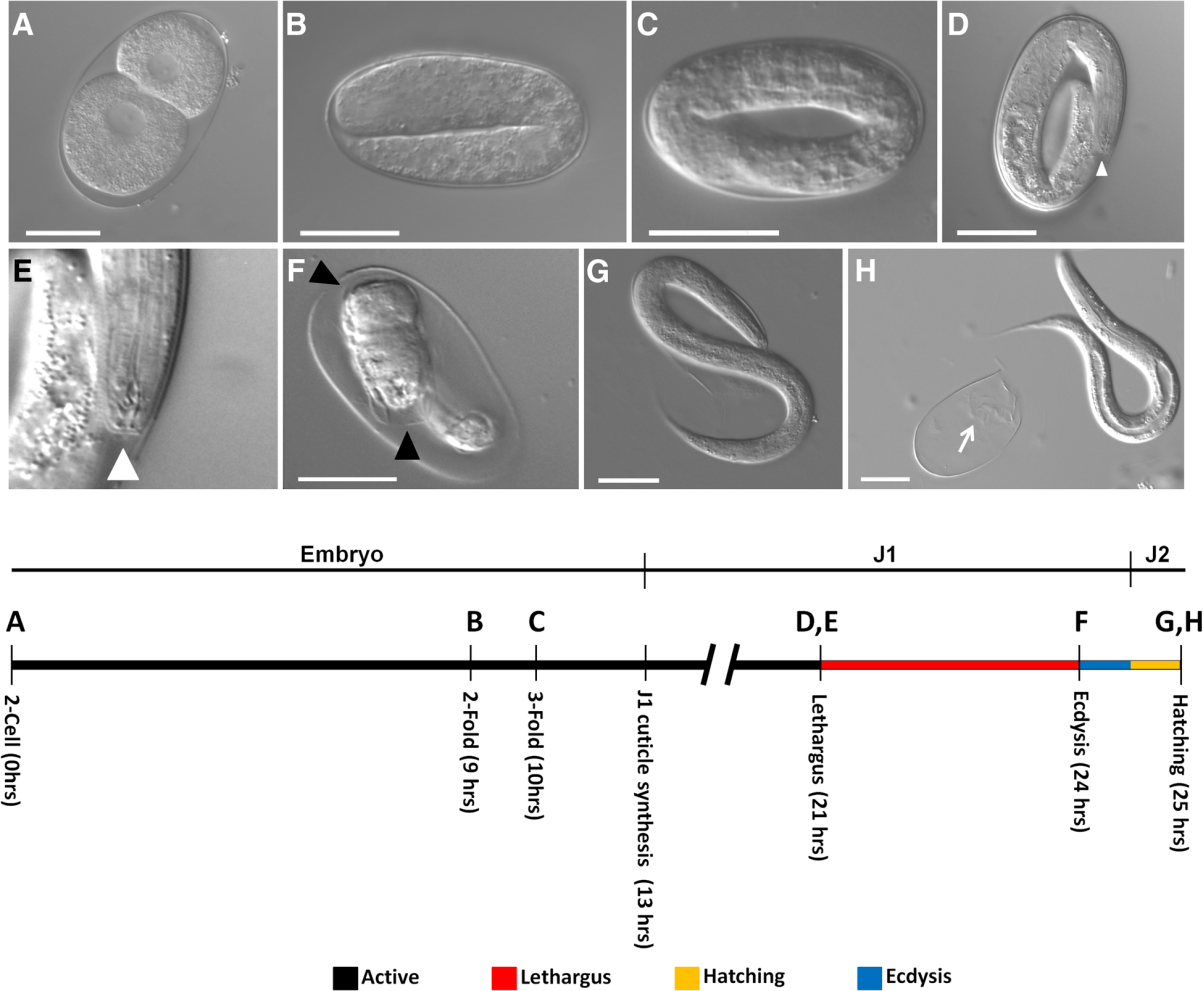
**A proposed sequence of *P. pacificus *pre-hatching development based on the current study and previously published data **[6]**,**[9]**.** Eggs are typically laid at the two-cell stage **(A)**. Elongation during late-stage embryogenesis includes 2-fold **(B)**, and 3-fold **(C)** embryos. Stage 1 juvenile (J1) cuticle synthesis is likely complete by the end of elongation (timing extrapolated from [8]) and marks the beginning of J1. Typical morphological features and associated behaviors found in L1 *C. elegans* occur in the pre-hatching J1 *P. pacificus.* Lethargus is defined by a lack of movement and formation of a buccal cap (**D**, **E**, white arrowhead) and is followed by ecdysis **(F)**. Black arrowheads show J1 cuticle just before ecdysis in *Ppa-mlt(csu28)***(F)**. Worms reach the second juvenile stage just prior to hatching **(G**, **H)**. White arrow **(H)** shows the remains of the J1 cuticle. Scale bars = 25 μm.

We reveal for the first time the behavioral and gene expression events associated with the first molt inside the eggshell of *P. pacificus*. Our findings show the highly stereotypical lethargus behavior occurring in the pre-hatching molt as well as the J2 to J3 molt in *P. pacificus,* and support the hypothesis that the pre-hatching molt is homologous to the post-hatching molts in *P. pacificus* and *C. elegans*. The newly isolated *Ppa-mlt* mutants show molting defects both before and after hatching, indicating that the mutations are required for proper molting in all developmental stages. Upregulation of *Ppa-pnhr-1* in pre-hatching J1 worms further corroborated our analysis. Interestingly, no homolog of *Ppa-pnhr-1*/Usp has been found in *C. elegans* and the functional role of *Ppa-pnhr-1* has not been determined. We summarize our results with previously published data to offer a consensus model of pre-hatching development in *P. pacificus*[6,10] (Figure 4)*.* Our study supports the hypothesis that the pre-hatching molt in *P. pacificus* is likely due to the result of a heterochronic shift in juvenile development and the timing of hatching [10].

The occurrence of the first molt prior to hatching in diverse plant and animal parasitic nematodes suggests multiple independent convergence events. One explanation may be that prolonging pre-hatching juvenile development before the infective dauer stage would provide selective advantage to parasitic nematodes. Alternatively, the co-occurrence of mouth dimorphism and pre-hatching molt in several genera within the Diplogastridae suggests that replacing the pharyngeal cuticle prior to hatching allows for the elaboration of the pharynx as a feeding organ during the course of evolution [10]. As J1 larvae are viable without the eggshell, identification of earlier hatching mutants may be a productive approach toward addressing the consequences of hatching as J1 larvae in *P. pacificus.*

Finally, we propose that the term, embryonic molt, be redefined as the pre-hatching molt in Diplogastrid nematodes, based on our observation that the pre-hatching molt occurs in the J1 to J2 transition just prior to hatching, when embryogenesis has already been completed. The results of our experiments have further characterized the pre-hatching development in *P. pacificus* with precise timing of lethargus as well as expression of *Ppa-pnhr-1* to better define genes associated with the first molt. These findings promote future studies on the genetic mechanisms responsible for the identified heterochronic changes in the timing of *P. pacificus* hatching, and may help to answer both how novelty occurs in developmental timing and how the presence of a pre-hatching developmental stage might contribute to the evolution of parasitism and the Diplogastrid dimorphic mouth formation.

### Materials and methods

#### Nematode and mutagenesis

*P. pacificus* wild-type and *emd* mutant lines from the PS312 background were cultured at room temperature (approximately 23°C) on *Escherichia coli* OP50 on nematode growth media [19]. Eggs were cultured at 20°C from synchronization until observation. Mutagenized strains were generated in the PS312 CA background using 50 mM ENU; 1,500 F1 animals were cloned and their F2 progeny inspected for the presence of molting defects. From plates containing mutants, defective animals were picked again and screened once more for the mutant phenotype. Mutant lines were backcrossed three times to wild-type PS312 and homozygous molting-defect escapers were established for detailed analyses.

#### Quantitative real-time PCR (qPCR)

J2 larvae were synchronized at the J2 arrest by bleaching gravid hermaphrodites and allowing the remaining embryos to hatch on unseeded plates for 12 hours. Eggs were synchronized by allowing young adult hermaphrodites to lay eggs for 15 minutes on plates seeded with OP50 prior to removal. cDNA was synthesized from RNA in Trizol with Superscript III First-Strand Synthesis Kit (Invitrogen, Calrsbad, California). qPCR was performed and analyzed using SYBR chemistry on BioRad CFX96 (Hercules, California) with primers designed to span exon-intron boundaries (forward, reverse): *Ppa-beta-tubulin*, TCCAAGATCCGTGAGGAGTA, GGAGAGGGTGGCATTGTAG; *Ppa-pnhr-1*, CTCTTGAACGGCGTCCCTCTTC, GTGCAGAGTTGCGAAGGCTG. qPCR data represent four technical replicates from two independently isolated RNA samples at each time point. Each biological replicate was collected from four pooled synchronized populations. Relative expression ratio of *Ppa-pnhr-1* to the reference gene *Ppa-beta-tubulin* was normalized to the no-reverse transcriptase negative control. Analysis of variance (ANOVA) was followed by the Dunn’s test.

#### Imaging

Worms were observed using either a Leica S8E stereomicroscope or a Leica DM6000 (Solms, Germany) for DIC imaging and prepared using Adobe Photoshop (San Jose, California). Eggs were mounted on slides with egg buffer without anesthetics for observation of the J1 larvae. For post-hatching experimentation, worms were observed on NGM culture plates at 80× with a Leica S8E dissecting microscope. For imaging, hatched juveniles were paralyzed with 40 μM sodium azide and mounted on slides with M9 buffer. Visual tracking of lethargus sequences in *P. pacificus* were made by observing synchronized embryos for 10 minutes every hour from 16 hours after egg laying until hatching. *C. elegans* embryos were tracked for 10 minutes every 30 minutes from 30 minutes after egg laying until hatching. Tracking of time until adulthood in *Ppa-mlt* alleles excludes those individuals that exhibited obvious molting defects. To visualize the presence of the pre-hatching molt in the mutants, worms from these lines were synchronized by egg-laying. When approximately 80% of larvae had hatched we screened the remaining eggs for pre-hatching juveniles that had not properly completed the molt.

#### Statistical analysis and graphical representations

Statistical analysis was performed using Excel (Microsoft, Redmond, Washington) and Instat software (GraphPad Software, San Diego, California). Graphical representations of collected data were prepared using Microsoft Excel, PowerPoint, and Adobe Photoshop.

## Abbreviations

J: juvenile stage; Usp: ultraspiracle.

## Competing interests

The authors declare that they have no competing interests.

## Authors’ contributions

Experimental design: RLH and VML; performed experiments: VML; data analysis: RLH and VML; prepared manuscript: RLH and VML. Both authors read and approved the final manuscript.

## Supplementary Material

Additional file 1 ***P. pacificus *** **active behavior.** DIC video of stage 1 juvenile (J1) *P. pacificus* PS312 wildtype during active behavior. Time-lapse video compiled from images taken over a 1.5 hour timeframe approximately 6 hours prior to hatching. Click here for file

Additional file 2 ***P. pacificus *** **lethargus behavior.** DIC video of stage 1 juvenile (J1) *P. pacificus* PS312 wildtype during lethargus behavior. Time-lapse video compiled from images taken over a 1.5 hour timeframe approximately 3 hours prior to hatching. Click here for file

Additional file 3 ***C. elegans *** **active behavior.** DIC video of *C. elegans* N2 wildtype embryo during active behavior. Time-lapse video compiled from images taken over a 1-hour timeframe approximately 3 hours prior to hatching. Click here for file

Additional file 4 ***Ppa-mlt(csu28) *** **mutant molting phenotype.** DIC Z-stack of the *Ppa-mlt(csu28)* molting deficiency in the stage 1 juvenile (J1) pre-hatching stage. Click here for file
